# Evolution of Hemophilia Management in Italy: Results from a Delphi Consensus Study

**DOI:** 10.3390/jcm15145741

**Published:** 2026-07-22

**Authors:** Giancarlo Castaman, Raimondo De Cristofaro, Matteo Nicola Dario Di Minno, Francesca Gatto, Paolo Mariani, Angelo Claudio Molinari, Mariasanta Napolitano, Cristina Santoro, Rita Carlotta Santoro, Ezio Zanon

**Affiliations:** 1 Department of Heart, Lung and Vessels, Center for Bleeding Disorders and Coagulation, Careggi University Hospital, 50134 Florence, Italy; castaman@aou-careggi.toscana.it; 2 Center for Hemorrhagic and Thrombotic Diseases, Foundation University Hospital “A. Gemelli” IRCCS, 00168 Rome, Italy; raimondo.decristofaro@unicatt.it; 3 Department of Clinical Medicine and Surgery, Federico II University of Naples, 80131 Naples, Italy; dario.diminno@hotmail.it; 4 Medical Affairs, Pfizer Italia, 00188 Rome, Italy; 5 Bicocca Applied Statistics Center (B-ASC), Department of Economics, Management and Statistics, University of Milan-Bicocca, 20126 Milan, Italy; paolo.mariani@unimib.it; 6 Thrombosis and Hemostasis Unit, IRCCS Istituto Giannina Gaslini, 16147 Genoa, Italy; aclaudiomolinari@gaslini.org; 7 Department of Health Promotion, Mother and Child Care, Internal Medicine and Medical Specialties (PROMISE), University of Palermo, 90127 Palermo, Italy; mariasanta.napolitano@unipa.it; 8 Haematology, Department of Translational and Precision Medicine, “Sapienza” University, 00161 Rome, Italy; santoro@bce.uniroma1.it; 9 Hemostasis and Thrombosis Unit, Hemato-Oncology Department, Azienda Ospedaliero-Universitaria Renato Dulbecco, 88100 Catanzaro, Italy; ritacarlottasantoro@gmail.com; 10 Haemophilia Center, First Chair of Internal Medicine, Padua University Hospital, 35128 Padua, Italy; ezio.zanon@unipd.it

**Keywords:** adherence, hemophilia, Delphi consensus, personalized therapy, prophylaxis, multidisciplinary care, PWH, joint ultrasound

## Abstract

**Background/Objectives**: The clinical management of hemophilia has evolved with the introduction of innovative therapies that are reshaping treatment paradigms. Real-world implementation of these approaches may vary across centers. This modified Delphi consensus study aimed to explore current clinical perspectives on hemophilia management in Italy among healthcare professionals. **Methods**: Eight hemophilia experts developed 38 statements covering seven domains, including therapeutic personalization, adherence and patient engagement, follow-up strategies, inhibitor management, comorbidity management in patients, musculoskeletal monitoring, and organizational aspects of care. Statements were evaluated through an anonymous web-based survey in a modified single-round Delphi process using a 5-point Likert scale. Consensus was defined a priori as ≥70% agreement or disagreement after exclusion of neutral responses. **Results**: Thirty-four clinicians from specialized hemophilia centers participated in the survey. Strong agreement emerged regarding the importance of personalized treatment strategies, active patient involvement in therapeutic decisions, and multidisciplinary management, particularly in ageing patients with comorbidities. Panelists also supported the role of joint ultrasound in monitoring musculoskeletal health and highlighted adherence as a key determinant of treatment outcomes. Areas of heterogeneity included criteria for transitioning between intravenous and subcutaneous therapies, the implementation of structured transition programs from pediatric to adult care, and the frequency of musculoskeletal imaging. Limited availability of psychological support and variable integration of patient-reported outcomes were also identified. **Conclusions**: Italian hemophilia specialists showed substantial agreement across key domains; however, future work should address remaining organizational and clinical gaps and incorporate patient perspectives to support more integrated and patient-centered management strategies for people with hemophilia.

## 1. Introduction

Hemophilia is an inherited X-linked bleeding disorder caused by a defect in the synthesis and/or function of factor VIII (FVIII) (hemophilia A) or factor IX (FIX) (hemophilia B), affecting the intrinsic pathway of coagulation [1]. People with hemophilia (PWH) usually experience bleeding episodes, either spontaneous or following injury or surgery. In particular, in severe cases, bleeding can also occur spontaneously, mostly into joints, potentially leading to chronic arthropathy [2,3]. Chronic pain and muscle atrophy in the peri-articular areas impact daily activities and patients’ quality of life (QoL) [4]. According to the Italian National Registry of Congenital Coagulopathies, more than 1500 patients are affected by severe hemophilia in Italy, 83.6% with severe hemophilia A and 16.4% with severe hemophilia B, although these proportions may be underestimated [5].

Standard treatment involves replacement of the missing clotting factor (VIII or IX), administered intravenously either on demand or as prophylaxis, depending on disease severity and bleeding phenotype [3]. Despite their efficacy [6], significant challenges remain, including potential subclinical bleeding risk, frequent intravenous infusions, inhibitor development, and chronic synovitis [2,3]. New and emerging therapeutic strategies, including extended half-life products, non-factor therapies, as well as rebalancing agents, and gene therapy [7], aim to reduce these complications [2,8] by improving adherence and joint health as well as extending life expectancy and quality of life in PWH [2,9]. As a result, PWH are increasingly ageing, with an increasing prevalence of both hemophilia-related complications (arthropathy, chronic viral infections) and age-associated conditions, including cardiovascular disease, cancer, and neurodegenerative disorders [8,9,10,11]. The management of these individuals is complex, and while many studies highlight the importance of a multidisciplinary approach, its implementation in routine clinical practice remains limited [2,11]. In Italy, this is reflected by substantial heterogeneity in care pathways and follow-up strategies [12].

Moreover, the generation of long-term clinical data on emerging therapies is important and needed to inform personalized treatment strategies, enable structured longitudinal follow-up, and support coordinated multidisciplinary management. Such evidence is also critical for guiding clinical decision-making, and treatment planning as well as monitoring; however, several studies have highlighted the limited availability of these data [2,3,13]. In this context, expert consensus may help address areas of uncertainty in clinical practice.

This modified Delphi study aims to gather real-world insights into the current landscape of hemophilia management in Italy, focusing on therapeutic personalization, structured follow-up, multidisciplinary care, and organizational aspects across the PWH lifespan, to identify areas of consensus and unmet needs in contemporary clinical practice.

## 2. Methods

### 2.1. The Delphi Method

The Delphi method is a widely used consensus approach in clinical research to address areas of uncertainty or limited evidence. Core methodological features include expert anonymity, structured rating, predefined consensus criteria, and, in some designs, iterative rounds with controlled feedback. Recognized reporting standards described in the literature for conducting and presenting the Delphi were followed in this work [14,15,16,17]. The participating panel was selected to gather insights from a group of experts specialized in hemophilia treatment and management.

### 2.2. Steering Committee and Statement Development

A steering committee composed of eight hematologists was established. Members were selected based on their expertise in clinical practice, roles as opinion leaders, and active involvement in relevant research activities. With the support of a methodologist, the steering committee convened an online meeting held on 18 June 2025 to identify priority topics to be addressed through the Delphi process. During the meeting, the Steering Committee identified priority domains based on current clinical challenges, areas of organizational variability, and unresolved issues in hemophilia management across Italian centers. A non-systematic literature search was conducted in PubMed to identify available evidence, previous consensus documents, and knowledge gaps relevant to these domains. Search terms included combinations of “hemophilia”, “prophylaxis”, “adherence”, “inhibitors”, “immune tolerance induction”, “musculoskeletal management”, “quality of life”, and “multidisciplinary management”. Based on the selected topics and the evidence from the literature, the statements were drafted offline by the Steering Committee members and iteratively refined through discussion. The final version of the statements was reviewed and validated by the independent methodologist before dissemination to the voting panel.

### 2.3. Delphi Round

The statements developed by the steering committee were uploaded onto a web-based platform for the collection of anonymous votes. Each statement was rated on a 5-point Likert scale (1 = strongly agree; 2 = agree; 3 = neither agree nor disagree; 4 = disagree; 5 = strongly disagree).

The questionnaire was distributed to experts working in specialized hemophilia centers across Italy, who were invited to independently evaluate each statement within a predefined timeframe. The voting panel was identified pragmatically with the aim of ensuring broad geographical and clinical representation across Italy [18]. All hemophilia centers were identified by the Steering Committee, and one or two clinicians from each center were invited to participate. Members of the Steering Committee were included among the invited panelists and participated in the voting process. Panelists received individual email invitations detailing the study’s objectives and methods.

### 2.4. Data Collection and Analysis

In November 2025, the statements were submitted to the panelists. Participants who expressed disagreement with a statement could provide a supporting comment to justify their choice.

Data were collected at the end of the predefined voting period and analyzed using descriptive statistics. For each statement, the distribution of responses across the 5-point Likert scale was calculated. The proportions of agreement (scores 1–2) and disagreement (scores 4–5) were computed and expressed as percentages. For consensus assessment, neutral responses (score 3) were excluded from the denominator. Therefore, agreement percentages reflect the proportion of respondents expressing a position on the statement. Consensus was defined a priori as ≥70% of respondents indicating agreement/disagreement, after exclusion of neutral ratings. Statements not reaching the predefined consensus threshold were reviewed by the Steering Committee. The possibility of conducting an additional Delphi round was considered after evaluation of the first-round results.

## 3. Results

### 3.1. Voting Panel

Overall, 85 panelists were invited to rate the Delphi statements and 34 responded (40% response rate), with participation varying across statements. The centers represented by the panelists collectively provide care for approximately 2000 patients with hemophilia across all disease severities.

**Figure 1:**
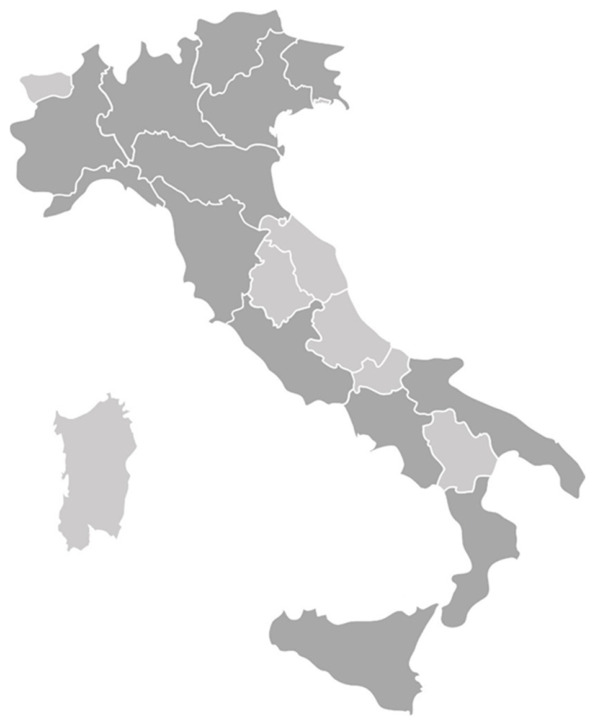
Geographical distribution of hemophilia centers participating in the modified Delphi panel across Italy. Regions with participating hemophilia centers are shaded in dark grey.

The panelists were hematologists working in specialized centers and managing both pediatric and adult PWH. The geographical distribution of the specialized centers involved is reported in Figure 1.

### 3.2. Delphi Process

A total of 38 statements, divided into seven domains, were evaluated through the Delphi process. The different domains reflected different thematic areas of hemophilia management, namely, therapeutic personalization and treatment selection, adherence and patient engagement, follow-up strategies and monitoring, management of inhibitors, management of comorbidities in older PWH, musculoskeletal assessment and imaging, and multidisciplinary and organizational aspects of care.

According to the predefined consensus criteria, 35 statements reached consensus within the first round. One item explored perceived barriers to the implementation of age-specific care pathways and was not rated with the Likert scale but required panelists to rank three predefined barriers according to their relevance. Given the overall level of agreement observed and the exploratory aim of the study, a second round was not undertaken, as areas of non-consensus were considered to reflect genuine heterogeneity in clinical practice rather than uncertainty related to statement wording.

Results are reported below according to the different domains. The complete response distribution for all statements is reported in Supplementary Tables S1–S7.

#### 3.2.1. Therapeutic Personalization and Treatment Selection

Table 1 reports the outcome for the first domain. Eight statements (1–8) reached the consensus threshold of ≥70% agreement, with five (statements 1, 2, 5, 6 and 7) reaching an agreement from all respondents to the specific statement (100%). The latter were related to the adoption of a personalized approach to long-term prophylaxis, the use of a multidimensional framework to assess clinical protection, the individualized criteria guiding therapeutic switches between factor and non-factor treatments, the role of subcutaneous administration in vulnerable populations, and the opportunity to initiate early management in infants through non-invasive therapeutic options (Table 1).

High levels of agreement were observed concerning the adequacy of currently available therapeutic options to support personalized care (statement 3, 96% agreement) and the existence of patient education strategies in the context of innovative subcutaneous therapies (statement 8, 86% agreement). Although consensus was achieved on the application of harmonized criteria for transitioning from intravenous to subcutaneous therapy (statement 4, 72% agreement), responses suggested more heterogeneity among centers.

The statement suggesting that therapeutic evolution may reduce the frequency of clinical assessments did not reach consensus (statement 9, 41% agreement).

#### 3.2.2. Adherence and Patient Engagement

Adherence to treatment and patient engagement in therapeutic management were evaluated by five statements and all reached agreement, with percentages ranging from 71% to 100% among those responding (Table 2).

The centrality of a relationship of trust and continuity between PWH and the clinical team, as well as the active involvement of PWH in therapeutic decision-making received unanimous consensus (statements 12 and 13, 100% agreement).

Panelists agreed that dosing frequency, treatment preparation, and route of administration are relevant barriers to adherence (statement 10, 86% agreement). Panelists also agreed on the fact that adherence is routinely monitored in Italian centers and is associated with clinical outcomes, including joint health (statement 11, 84% agreement).

The presence of structured transition pathways within single centers, involving coordinated interaction between pediatric and adult teams (statement 14) reported an agreement of 71%, with a substantial proportion of panelists opting for a neutral response.

**Figure 2:**
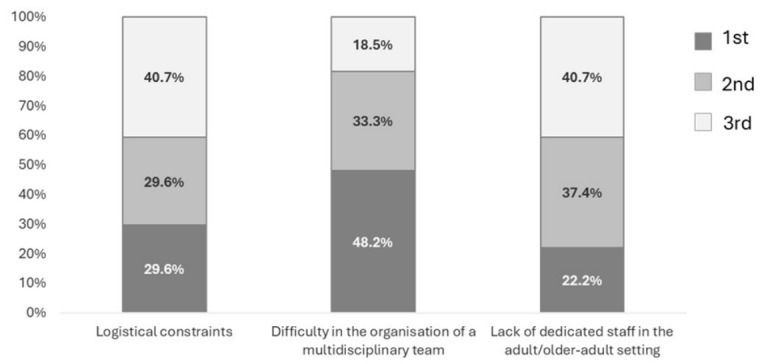
Ranking of the main organizational barriers to multidisciplinary care in the adult/older-adult setting. Stacked bar chart showing the ranking of key organizational barriers affecting the implementation of multidisciplinary care in the adult/older-adult clinical setting. Respondents ranked each barrier as first (1st), second (2nd), or third (3rd) in importance.

When asked to rank the main challenges in implementing age-specific care pathways, the difficulty in organizing a multidisciplinary team emerged as the prevalent barrier, being ranked as the primary challenge by 48.2% of the panelists (Figure 2). The lack of dedicated staff in adult or older-adult settings and logistical constraints was also identified as relevant obstacles, although they were more often ranked as second-level challenges (37.4% and 29.6%, respectively).

#### 3.2.3. Follow-Up Strategies and Monitoring

All four statements within this domain reached agreement (Table 3). Regular follow-up visits, potentially through the use of telemedicine, a trusting relationship with the hemophilia center, and the alignment of the type of treatment administration with patient preferences are key components supporting long-term adherence (statements 16 and 18, 90% and 100% agreement among respondents, respectively).

Among possible strategies to monitor follow-up, strong consensus emerged on the need to use systematic objective measures to assess treatment adherence, considering behavioral or psychosocial factors before modifying therapy when suboptimal response to prophylaxis is reported (statement 19, 90% agreement).

In case of poor clinical response while on prophylaxis, alternative regimens, such as on-demand treatment, may be considered, particularly in PWH experiencing a high treatment burden or difficulties with treatment administration for 72% of the panelists (statement 17).

#### 3.2.4. Management of Inhibitors

The six statements within this domain reached a consistent agreement among respondents (Table 4). Unanimous consensus was observed regarding the importance of routine monitoring of inhibitors in PWH A with Bethesda or Nijmegen assays, the need for inhibitor testing in hemophilia B in the presence of unexplained allergic reactions, and the fact that complete immune tolerance induction (ITI) success needs to be defined according to standardized criteria (statements 20, 21, and 25, 100% agreement).

Strong agreement was also reached on the need for a case-by-case evaluation of inhibitor eradication in PWH A receiving non-factor prophylaxis, taking into account potential future need of factor VIII use (statement 22, 96% agreement). Similarly, 95% of panelists would consider inhibitor eradication in light of the possible extra-hemostatic biological effects of FVIII, mainly in preserving bone health (statement 23).

ITI in people with hemophilia B should be provided only in specialized centers, for 96% of panelists (statement 24).

#### 3.2.5. Management of Comorbidities in Older People with Hemophilia

Both statements within this domain reached agreement (Table 5). Unanimous agreement was expressed on the importance of a multidisciplinary approach in the management of older or comorbid PWH (statement 26, 100% agreement considering the respondents to this statement). The routine assessment of thrombotic risk considering patient age and family history, comorbidities (mainly cardiovascular), and therapeutic choices to support informed treatment decisions is also acknowledged in Italian centers by 87% of the panelists (statement 27).

#### 3.2.6. Musculoskeletal Assessment and Imaging

Four of the five statements (28, 29, 31, 32) of this domain reached the consensus threshold (Table 6). Panelists agreed on the role of joint ultrasound in the follow-up of PWH, either as part of regular monitoring or when clinically indicated by new symptoms, suspected hemarthrosis, functional impairment, or therapeutic changes (statements 28 and 29, 86% and 85% agreement, respectively).

Moreover, high agreement levels were also observed regarding the use of additional imaging modalities in case ultrasonographic findings are inconclusive (statement 31, 96% agreement), as well as performing short-interval ultrasound reassessment followed by further imaging if clinical–imaging discordance is observed (statement 32, 93% agreement).

In contrast, there was no agreement on the existence of defined and shared criteria to determine the frequency of joint ultrasound examinations in the Italian centers (statement 30, 50% agreement). This statement is also characterized by an elevated number of neutral responses.

#### 3.2.7. Multidisciplinary and Organizational Aspects of Care

General agreement was observed across several aspects related to the multidisciplinary and organizational management of hemophilia care, although agreement rates were overall lower than those reported in other domains (Table 7). Panelists agreed that patient-reported outcomes are routinely investigated in clinical practice to support a comprehensive patient-centered approach (statement 33, 85% agreement).

An agreement of 72% on the lack of psychological counselling across hemophilia centers was reported (statement 34).

Genetic and reproductive counselling for women carrying hemophilia-causing gene variants and for families of pediatric patients is available in Italian centers (statement 35, 73% agreement), as well as the screening for inherited coagulation disorders in women presenting with unexplained abnormal uterine bleeding (statement 36, 75% agreement). Unanimous agreement was observed on the need to measure FVIII and FIX levels in women who are asymptomatic carriers or suspected carriers of hemophilia-causing gene variants (statement 37, 100% agreement). Ninety-one percent of panelists also agreed that educational counselling programs for PWH, their caregivers and families are an integral component of care in Italian hemophilia centers (statement 38).

## 4. Discussion

This modified Delphi study explored current clinical practices and healthcare professional perspectives on hemophilia management in Italy.

Overall, a high level of consensus was observed across most of the statements, reflecting a shared vision among Italian centers regarding the evolution of care. At the same time, some areas of heterogeneity emerged, highlighting organizational challenges and unmet needs that may inform future clinical and organizational strategies and interventions.

One of the most consistent observations was the strong agreement on personalized therapeutic strategies, reflecting the increasing recognition that hemophilia management should be individualized according to clinical and bleeding phenotype, patient characteristics, lifestyle factors, and treatment preferences [19,20].

The consensus regarding the role of subcutaneous therapies in vulnerable populations supports the growing attention dedicated to new therapeutic strategies to address individual patient needs. Nevertheless, the marginal agreement reached on the existence of harmonized criteria for transitioning from intravenous to subcutaneous therapy in long-term prophylaxis suggests heterogeneity among centers. Future consensus initiatives should address elements to be included in a shared “minimum dataset” to support treatment decisions (e.g., bleeding phenotype, venous access, age, patient preferences, comorbidities, inhibitor status).

While new therapeutic options may reduce treatment burden and often decrease bleeding frequency, this does not translate into a reduction in clinical monitoring, which remains essential for the follow-up of PWH. Guideline recommendations for regular multidisciplinary assessments and monitoring of treatment outcomes apply regardless of the therapy used [6]. The nature of follow-up may evolve rather than diminish, with monitoring increasingly including assessment of adherence, patient-reported outcomes, pain evaluation, joint health, thrombotic risk, and comorbidities, rather than focusing exclusively on bleeding events [6,21,22]. In this context, hybrid follow-up models integrating telemedicine [23] with in-person evaluations may represent a promising approach to maintain continuity of care while adapting to changing treatment paradigms, especially in individuals with suboptimal clinical response or adherence issues. Although validated questionnaires for capturing patient-reported outcomes are available [24,25], their routine use during follow-up visits may be limited by time constraints. The integration of telemedicine tools may help enable the collection of these data remotely, thereby facilitating a more comprehensive assessment of patient-reported outcomes between scheduled visits.

Adherence to prophylaxis remains a key determinant of clinical outcomes in hemophilia, with treatment burden representing one of the main barriers [26], particularly among adolescents and young adults [27,28]. Given that effective transition programs between pediatric and adult care are important determinants of long-term outcomes [29], this area may represent a relevant target for organizational improvement in Italian centers. Where pediatric and adult services operate within the same clinical unit, the transition process may be facilitated. Conversely, in centers where pediatric and adult care are not integrated, the implementation of multidisciplinary collaborations, including regular meetings among teams and gradual handover processes, can ensure continuity of care.

Treatment adherence can be negatively affected by difficulties with venous access, reluctance to maintain regular intravenous therapy, and logistical barriers. The switch to on-demand regimens in these situations represents a critical concern. Addressing the determinants of suboptimal adherence by reducing treatment burden may be the best option impacting on clinical outcomes. A stepwise approach with an objective assessment of adherence and the evaluation of potential behavioral or psychosocial factors should be undertaken before considering any modification of the therapeutic strategy [30,31].

While inhibitor monitoring should be routinely performed, eradication in PWH receiving prophylaxis with non-factor therapies should be carefully individualized. Indeed, the presence of an inhibitor may still represent a clinical limitation in scenarios where factor replacement therapy may be required, such as major surgery, trauma, or other acute complications. Therefore, the potential future need for FVIII or FIX should be considered when evaluating the appropriateness of eradicating the inhibitor with ITI [2].

The increasing life expectancy of PWH has resulted in a growing population of older PWH with multiple comorbidities [8]. In line with this epidemiological shift, a multidisciplinary approach involving specialists managing co-existing conditions is required. The routine assessment of thrombotic risk emerged as an important component of clinical practice in this group, suggesting the systematic evaluation of this risk as a central and timely issue in hemophilia care [32]. The evaluation of pro-thrombotic risk with tools used in the general population, integrated with hemophilia-specific considerations, should be considered. These findings highlight the need for practical approaches to risk stratification and its regular reassessment, and for clear strategies to manage intercurrent bleeding events in those who may simultaneously present prothrombotic risk factors.

Musculoskeletal health remains a cornerstone of hemophilia management. Panelists strongly supported the role of joint ultrasound as a key tool for the early detection of joint abnormalities and for monitoring joint health during follow-up [33]. However, both routine and indication-based ultrasound strategies have met the agreement of the experts, suggesting that centers currently adopt heterogeneous monitoring approaches, likely due to a lack of shared national criteria for the frequency of such examinations. The systematic use of ultrasound at every follow-up visit may be limited by the availability of trained personnel, dedicated time, and technical resources. Shared monitoring strategies, possibly with the use of validated scores, emerged as a clinical need to guide musculoskeletal follow-up across hemophilia centers. Such approaches should consider sustainability and appropriateness of resource utilization, potentially adopting monitoring strategies based on individual patient characteristics, joint status, and treatment regimen [6,33].

The modified Delphi highlighted several organizational aspects that remain heterogeneous across Italian hemophilia centers: systematic integration of patient-reported outcomes into routine practice, limited availability of psychological support despite its potential relevance in addressing treatment-related anxiety, chronic pain, and quality-of-life issues, or the availability of genetic and reproductive counselling for women carrying hemophilia mutations. Strengthening collaboration between hemophilia centers and specialists in obstetrics and gynecology may represent an important area for future development.

### Strengths and Limitations

This study presents a comprehensive overview of current clinical hemophilia management in Italy from the healthcare professional perspective, providing expert opinions on existing clinical gaps. However, some limitations should be acknowledged. First, the study relied on expert opinion and therefore reflects perceptions of current practice in specialized centers, which limits the generalizability of the findings. Despite the high level of agreement observed across many statements, these findings should be interpreted with caution, as the observed consensus may partly reflect the composition of the panel and the methodological approach adopted, rather than a fully homogeneous clinical practice across all Italian centers. It should be noted that the members of the Steering Committee also participated in the voting process. This is an approach commonly adopted in Delphi studies involving rare diseases, where the contribution of highly experienced experts is essential, although the involvement of some panelists in both statement development and voting should be considered when interpreting the findings. Notably, areas characterized by residual uncertainty or variability, such as criteria for therapeutic switching, transition pathways, and musculoskeletal monitoring, may be more informative in identifying gaps in care than statements with near-universal agreement. In addition, the variability in the number of responses across statements may reflect differences in engagement with specific topics. Since agreement percentages were calculated after excluding neutral responses, they reflect the level of agreement among respondents expressing a position on each statement and the full distribution of responses should be taken into account. As participation in the modified Delphi survey was voluntary, a degree of selection bias cannot be excluded. Although the overall response rate was 40%, response rates in Delphi studies are variable and no universally accepted minimum response-rate threshold has been established to define methodological adequacy [34]. Furthermore, Delphi methodology is a qualitative, expert-based consensus approach aimed at exploring and synthesizing opinions and levels of agreement among selected experts rather than generating statistically representative estimates of a broader population. Therefore, the findings should be interpreted as reflecting the views and consensus achieved within the participating expert panel. Since the reasons for non-participation were not collected, it was not possible to assess whether non-respondents differed systematically from respondents. Nevertheless, the participating clinicians represented the hemophilia centers distributed throughout Italy, providing broad geographical and clinical representation. Moreover, the panel did not involve people with hemophilia; therefore the findings reflect only clinician perspectives and should not be interpreted as representative of patient experiences, preferences, or patient-reported priorities. Future studies specifically designed to optimize care pathways should incorporate patient representation to complement the professional perspective. Despite the Delphi technique involving iterative rounds, in this study the single round limited the possibility to reconsider the responses after aggregated feedback from the whole panel. However, modified Delphi approaches with a single round have been reported in the literature [35,36,37], particularly when the objective is to obtain an overview of expert perspectives. The high level of agreement observed for many statements likely reflects the consolidated management of hemophilia within Italian specialized centers. In this sense, the Delphi process allowed confirmation of widely shared clinical practices while also identifying specific areas where heterogeneity or organizational gaps persist.

## 5. Conclusions

This study highlights broad consensus among Italian hemophilia specialists regarding personalized treatment strategies, patient engagement, and multidisciplinary care. However, several unmet needs remain, particularly the lack of shared criteria for transitioning between intravenous and subcutaneous therapies, variability in transition pathways from pediatric to adult care, and the absence of harmonized protocols for musculoskeletal monitoring. Additional gaps emerged in the systematic integration of patient-reported outcomes into clinical routine, access to psychological support, and the practical management of thrombotic risk in ageing patients. Addressing these gaps may support the development of more integrated care models capable of responding to the evolving needs of PWH.

## Figures and Tables

**Table 1:** Expert agreement on therapeutic personalization and treatment selection. N = number of voting panelists after excluding neutral responses.

Statement	% Agreement	N
1. In Italian centers, the choice of therapy for long-term prophylaxis is guided by a personalized approach (patient age, bleeding phenotype, lifestyle, expected adherence, individual patient preferences, and route of administration).	100	33
2. Clinical protection is assessed through a multidimensional approach that complements laboratory and joint-health assessment, using tools such as point-of-care ultrasound, HJHS or performance tests (e.g., 6-Minute Walking Test).	100	32
3. The availability of all therapeutic options (factor-based and non-factor-based) in Italian centers is currently adequate to ensure a personalized approach	96	28
4. Italian centers apply harmonized criteria for transitioning from intravenous to subcutaneous therapy in long-term prophylaxis.	72	18
5. The switch from factor replacement therapy to a non-factor therapy (or vice versa) is based on individual clinical data and the available evidence on long-term efficacy and safety.	100	28
6. In pediatric and elderly patients, presenting with specific conditions such as compromised venous access, reduced manual dexterity, physical frailty, cognitive impairment or decline, or social isolation, subcutaneous administration ensures continuity of treatment, independence, and adherence.	100	30
7. In infants with hemophilia, the availability of non-invasive therapies enables initiation of care immediately after diagnosis, improving clinical management and allowing individualized treatment.	100	29
8. In patients receiving innovative subcutaneously administered therapies, centers calibrate the scope of information and education to the reduced frequency of clinic visits, prioritizing treatment self-management and the recognition of bleeding symptoms.	86	22
9. The evolution of therapies may lead to a reduction in the frequency of clinical assessments in patients treated for hemophilia.	41	22

**Table 2:** Expert agreement on treatment adherence and transition of care. N = number of voting panelists after excluding neutral responses.

Statement	% Agreement	N
10. Dosing frequency, treatment preparation, and administration route are among the main barriers to adherence in patients with hemophilia.	86	22
11. Adherence is tracked in Italian centers, and the results correlate with clinical outcomes, including joint health.	84	19
12. A relationship of trust and continuity with the clinical team is fostered to achieve greater adherence to prophylaxis	100	28
13. In Italian centers, patients are actively involved in therapeutic decision-making to strengthen motivation for proactive self-management and to improve adherence to prophylaxis.	100	28
14. In Italian practice, specific programs for adolescents and adults operate within a single center, with structured, regular meetings between the pediatric and adult teams to facilitate clinician handover and to introduce patients to the new team that will be in charge of their care.	71	14

**Table 3:** Expert agreement on statements related to follow-up strategies and patient monitoring. N = number of voting panelists after excluding neutral responses.

Statement	% Agreement	N
16. In patients reporting a poor clinical response, regular visits and a trusting relationship with the hemophilia center, supported by telemedicine, are integral components of the therapeutic strategy to promote better adherence.	90	20
17. In patients with poor clinical response while on prophylaxis, the use of on-demand regimens or partial prophylaxis (one infusion per week) is considered in cases of documented poor adherence, high treatment burden, or administration difficulties.	72	18
18. Aligning treatment administration type with patient preferences and needs supports better long-term adherence.	100	27
19. In routine clinical practice in Italy, for patients showing a suboptimal response to prophylaxis, treatment adherence is systematically assessed using objective measures, and potential behavioral or psychosocial factors are considered before making any treatment changes.	90	20

**Table 4:** Expert agreement on statements related to inhibitor management in hemophilia. N = number of voting panelists after excluding neutral responses.

Statement	% Agreement	N
20. In patients with hemophilia A, inhibitor monitoring using the Bethesda or Nijmegen assay is essential to determine the optimal therapeutic strategy and to detect early changes in response, and is routinely performed across all centers.	100	29
21. Given the biological features of hemophilia B, testing for inhibitors is indicated not only in cases of loss of hemostatic response but also when unexplained allergic reactions occur.	100	28
22. In patients with hemophilia A and inhibitor, receiving prophylaxis with non-factor therapies, the decision to pursue inhibitor eradication should be made case by case, considering potential future needs for factor VIII in emergency situations (e.g., major surgery, trauma, complications) that may require faster-acting agents for acute management.	96	27
23. The presence of inhibitors in hemophilia A may potentially abolish residual biological effects of factor VIII on other pathways (e.g., bone remodeling); therefore, the possibility to pursue complete inhibitor eradication even during non-factor therapy should be considered based on the possible extra-hemostatic effects.	95	19
24. Only specialized centers deliver ITI for hemophilia B, with close monitoring and shared decision-making with the patient and/or their family.	96	23
25. Within Italian clinical practice, complete success of ITI is defined according to standardized, consensus-derived criteria.	100	25

**Table 5:** Expert agreement on the management of comorbidities in older people with hemophilia. N = number of voting panelists after excluding neutral responses.

Statement	% Agreement	N
26. The management of older or comorbid patients with hemophilia should be multidisciplinary, with structured coordination between specialists responsible for their coexisting conditions and therapies.	100	29
27. In Italian centers, thrombotic risk is assessed routinely, considering patient age, comorbidities, and decision to start a non-factor therapy, to inform treatment decisions.	87	23

**Table 6:** Expert agreement on statements related to musculoskeletal assessment and imaging in hemophilia. N = number of voting panelists after excluding neutral responses.

Statement	% Agreement	N
28. At every follow-up visit, joint ultrasonography should be performed to document joint status and to enable early detection of hemarthrosis or synovitis, regardless of the interval between visits.	86	22
29. Joint ultrasound is performed only when clinically indicated (new articular symptoms, suspected hemarthrosis, functional impairment, therapeutic changes); in the absence of such indications, a program of periodic imaging of the major joints at pre-established intervals is adopted.	85	27
30. Across all Italian centers there are defined and shared criteria for determining the frequency of joint ultrasound examinations during follow-up.	50	18
31. If a joint ultrasound does not provide a clear diagnosis, it is appropriate to use other imaging modalities (magnetic resonance imaging [MRI], computed tomography [CT], or plain X-rays) for further evaluation.	96	26
32. If an abnormal or equivocal ultrasonographic finding is observed during follow-up, a short-interval ultrasound re-evaluation is advisable; should the abnormality persist, or if there is clinical–imaging discordance, the use of other imaging modalities (MRI, CT, radiography) is indicated.	93	27

**Table 7:** Expert agreement on statements related to multidisciplinary and organizational aspects of hemophilia care. N = number of voting panelists after excluding neutral responses.

Statement	% Agreement	N
33. Physicians managing hemophilia assess and incorporate patient-reported outcomes–such as emotional well-being, acute and chronic pain, and quality of life–into routine practice to ensure a truly patient-centered approach.	85	26
34. Psychological counselling is available at hemophilia centers in Italy across all stages of the patient’s life, with particular attention to adolescence and the management of treatment-related anxiety.	28	18
35. Genetic and reproductive counselling is offered at every center to women carrying a hemophilia-causing mutation and to the families of pediatric patients, from the time of diagnosis.	73	22
36. In women with abnormal uterine bleeding without documented gynecologic causes, screening is performed for inherited coagulation defects and for hemophilia carrier status, even in the absence of a family history.	75	24
37. Measurement of FVIII and FIX levels should be performed in women who are asymptomatic carriers or suspected carriers.	100	29
38. In Italian hemophilia centers, educational counselling programs for patients, caregivers, and parents of pediatric patients are an integral part of care to enhance disease understanding, promote self-management, support safe home treatment, and improve family quality of life.	91	22

## Data Availability

All data generated in the work are included in the article.

## Supplementary Materials

The following supporting information can be downloaded at: https://www.mdpi.com/article/10.3390/jcm15145741/s1, Table S1. Distribution of responses across Likert-scale categories for statements on therapeutic personalization and treatment selection. N = number of voting panelists. Table S2. Distribution of responses across Likert-scale categories for statements on treatment adherence and transition of care. N = number of voting panelists. Table S3. Distribution of responses across Likert-scale categories for statements related to follow-up strategies and patient monitoring. N = number of voting panelists. Table S4. Distribution of responses across Likert-scale categories for statements related to inhibitor management in hemophilia. N = number of voting panelists. Table S5. Distribution of responses across Likert-scale categories for statements on the management of comorbidities in older people with hemophilia. N = number of voting panelists. Table S6. Distribution of responses across Likert-scale categories for statements related to musculoskeletal assessment and imaging in hemophilia. N = number of voting panelists. Table S7. Distribution of responses across Likert-scale categories for statements related to patient-centered care, counselling, and women’s health. N = number of voting panelists.

## Abbreviations

The following abbreviations are used in this manuscript:

CT	Computed Tomography
FVIII/FIX	Factor VIII/Factor IX
HJHS	Haemophilia Joint Health Score
ITI	Immune Tolerance Induction
MRI	Magnetic Resonance Imaging
PWH	People with Hemophilia
QoL	Quality of Life
