# Supplementary material for: Evolution of Hemophilia Management in Italy: Results from a Delphi Consensus Study
Source: J Clin Med. 2026 Jul 22;15(14):5741. doi: 10.3390/jcm15145741 (PMC13412974; doi:10.3390/jcm15145741)
Supplement: Supplementary file 1 [file jcm-15-05741-s001.zip › jcm-4369216-supplementary.pdf]

Supplementary Materials

Evolution of Hemophilia Management in Italy: Results from a Delphi Consensus Study

Giancarlo Castaman, Raimondo De Cristofaro, Matteo Nicola Dario Di Minno, Francesca Gatto, Paolo Mariani, Angelo Claudio Molinari, Mariasanta Napolitano, Cristina Santoro, Rita Carlotta Santoro, Ezio Zanon

Table S1. Distribution of responses across Likert-scale categories for statements on therapeutic personalization and treatment selection. N = number of voting panelists.

Statement	Completely Agree, N (%)	Agree, N (%)	Neither Agree nor Disagree, N (%)	Disagree, N (%)	Completely Disagree, N (%)	N
1. In Italian centers, the choice of therapy for long-term prophylaxis is guided by a personalized approach (patient age, bleeding phenotype, lifestyle, expected adherence, individual patient preferences, and route of administration).	20 (59)	13 (38)	1 (3)	0	0	34
2. Clinical protection is assessed through a multidimensional approach that complements laboratory and joint-health assessment, using tools such as point-of-care ultrasound, HJHS or performance tests (e.g., 6-Minute Walking Test).	14 (42)	18 (55)	1 (3)	0	0	33
3. The availability of all therapeutic options (factor-based and non-factor-based) in Italian centers is currently adequate to ensure a personalized approach	9 (27)	18 (55)	5 (15)	1 (3)	0	33
4. Italian centers apply harmonized criteria for transitioning from intravenous to subcutaneous therapy in long-term prophylaxis.	1 (3)	12 (38)	14 (44)	5 (16)	0	32
5. The switch from factor replacement therapy to a non–factor therapy (or vice versa) is based on individual clinical data and the available evidence on long-term efficacy and safety.	8 (25)	20 (62.5)	4 (12.5)	0	0	32
6. In pediatric and elderly patients, presenting with specific conditions such as compromised venous access, reduced manual dexterity, physical frailty, cognitive impairment or decline, or social isolation, subcutaneous administration ensures continuity of treatment, independence, and adherence.	12 (39)	18 (58)	1 (3)	0	0	31
7. In infants with hemophilia, the availability of non-invasive therapies enables initiation of care immediately after	20 (65)	9 (29)	2 (6)	0	0	31

diagnosis, improving clinical management and allowing individualized treatment.						
8. In patients receiving innovative subcutaneously administered therapies, centers calibrate the scope of information and education to the reduced frequency of clinic visits, prioritizing treatment self-management and the recognition of bleeding symptoms.	4 (13)	15 (48)	9 (29)	3 (10)	0	31
9. The evolution of therapies may lead to a reduction in the frequency of clinical assessments in patients treated for hemophilia.	2 (6.5)	7 (22.6)	9 (29)	11 (35.4)	2 (6.5)	31

Table S2. Distribution of responses across Likert-scale categories for statements on treatment adherence and transition of care. N = number of voting panelists.

Statement	Completely Agree, N (%)	Agree, N (%)	Neither Agree nor Disagree, N (%)	Disagree, N (%)	Completely Disagree, N (%)	N
10. Dosing frequency, treatment preparation, and administration route are among the main barriers to adherence in patients with hemophilia.	7 (23)	12 (39)	9 (29)	3 (10)	0	31
11. Adherence is tracked in Italian centers, and the results correlate with clinical outcomes, including joint health.	1 (3)	15 (48)	12 (39)	3 (10)	0	31
12. A relationship of trust and continuity with the clinical team is fostered to achieve greater adherence to prophylaxis	13 (43)	15 (50)	2 (7)	0	0	30
13. In Italian centers, patients are actively involved in therapeutic decision-making to strengthen motivation for proactive self-management and to improve adherence to prophylaxis.	5 (16.7)	23 (76.6)	2 (6.7)	0	0	30
14. In Italian practice, specific programs for adolescents and adults operate within a single center, with structured, regular meetings between the pediatric and adult teams to facilitate clinician handover and to introduce patients to the new team that will be in charge of their care.	1 (3)	9 (31)	15 (52)	4 (14)	0	29

Table S3. Distribution of responses across Likert-scale categories for statements related to follow-up strategies and patient monitoring. N = number of voting panelists.

Statement	Completely Agree, N (%)	Agree, N (%)	Neither Agree nor Disagree, N (%)	Disagree, N (%)	Completely Disagree, N (%)	N
16. In patients reporting a poor clinical response, regular visits and a trusting relationship with the hemophilia center, supported by telemedicine, are integral components of the therapeutic strategy to promote better adherence.	2 (7)	16 (55)	9 (31)	2 (7)	0	29
17. In patients with poor clinical response while on prophylaxis, the use of on-demand regimens or partial prophylaxis (one infusion per week) is considered in cases of documented poor adherence, high treatment burden, or administration difficulties.	0	13 (45)	11 (38)	5 (17)	0	29
18. Aligning treatment administration type with patient preferences and needs supports better long-term adherence.	8 (28)	19 (65)	2 (7)	0	0	29
19. In routine clinical practice in Italy, for patients showing a suboptimal response to prophylaxis, treatment adherence is systematically assessed using objective measures, and potential behavioral or psychosocial factors are considered before making any treatment changes.	3 (10)	15 (52)	9 (31)	2 (7)	0	29

Table S4. Distribution of responses across Likert-scale categories for statements related to inhibitor management in hemophilia. N = number of voting panelists.

Statement	Completely Agree, N (%)	Agree, N (%)	Neither Agree nor Disagree, N (%)	Disagree, N (%)	Completely Disagree, N (%)	N
20. In patients with hemophilia A, inhibitor monitoring using the Bethesda or Nijmegen assay is essential to determine the optimal therapeutic strategy and to detect early changes in response, and is routinely performed across all centers.	15 (52)	14 (48)	0	0	0	29
21. Given the biological features of hemophilia B, testing for inhibitors is indicated not only in cases of loss of hemostatic response but also when unexplained allergic reactions occur.	17 (59)	11 (38)	1 (3)	0	0	29
22. In patients with hemophilia A and inhibitor, receiving prophylaxis with non-factor therapies, the decision to pursue inhibitor eradication should be made case by case, considering potential future needs for factor VIII in emergency situations (e.g., major surgery, trauma, complications) that may require faster-acting agents for acute management.	11 (38)	15 (52)	2 (7)	1 (3)	0	29
23. The presence of inhibitors in hemophilia A may potentially abolish residual biological effects of factor VIII on other pathways (e.g., bone remodeling); therefore, the possibility to pursue complete inhibitor eradication even during non–factor therapy should be considered based on the possible extra-hemostatic effects.	3 (10)	15 (52)	10 (35)	1 (3)	0	29
24. Only specialized centers deliver ITI for hemophilia B, with close monitoring and shared decision-making with the patient and/or their family.	10 (35)	12 (41)	6 (21)	1 (3)	0	29
25. Within Italian clinical practice, complete success of ITI is defined according to standardized, consensus-derived criteria.	12 (41)	13 (45)	4 (14)	0	0	29

Table S5. Distribution of responses across Likert-scale categories for statements on the management of comorbidities in older people with hemophilia. N = number of voting panelists.

Statement	Completely Agree, N (%)	Agree, N (%)	Neither Agree nor Disagree, N (%)	Disagree, N (%)	Completely Disagree, N (%)	N
26. The management of older or comorbid patients with hemophilia should be multidisciplinary, with structured coordination between specialists responsible for their coexisting conditions and therapies.	20 (69)	9 (31)	0	0	0	29
27. In Italian centers, thrombotic risk is assessed routinely, considering patient age, comorbidities, and decision to start a non-factor therapy, to inform treatment decisions.	2 (7)	18 (62)	6 (21)	3 (10)	0	29

Table S6. Distribution of responses across Likert-scale categories for statements related to musculoskeletal assessment and imaging in hemophilia. N = number of voting panelists.

Statement	Completely Agree, N (%)	Agree, N (%)	Neither Agree nor Disagree, N (%)	Disagree, N (%)	Completely Disagree, N (%)	N
28. At every follow-up visit, joint ultrasonography should be performed to document joint status and to enable early detection of hemarthrosis or synovitis, regardless of the interval between visits.	10 (35)	9 (31)	7 (24)	3 (10)	0	29
29. Joint ultrasound is performed only when clinically indicated (new articular symptoms, suspected hemarthrosis, functional impairment, therapeutic changes); in the absence of such indications, a program of periodic imaging of the major joints at pre-established intervals is adopted.	6 (21)	17 (58)	2 (7)	2 (7)	2 (7)	29
30. Across all Italian centers there are defined and shared criteria for determining the frequency of joint ultrasound examinations during follow-up.	0	9 (31)	11 (38)	8 (28)	1 (3)	29
31. If a joint ultrasound does not provide a clear diagnosis, it is appropriate to use other imaging modalities (MRI, CT, or plain X-rays) for further evaluation.	8 (27)	17 (59)	3 (10)	1 (4)	0	29
32. If an abnormal or equivocal ultrasonographic finding is observed during follow-up, a short-interval ultrasound re-evaluation is advisable; should the abnormality persist, or if there is clinical–imaging discordance, the use of other imaging modalities (MRI, CT, radiography) is indicated.	9 (31)	16 (55)	2 (7)	2 (7)	0	29

Table S7. Distribution of responses across Likert-scale categories for statements related to patient-centered care, counselling, and women's health. N = number of voting panelists.

Statement	Completely Agree, N (%)	Agree, N (%)	Neither Agree nor Disagree, N (%)	Disagree, N (%)	Completely Disagree, N (%)	N
33. Physicians managing hemophilia assess and incorporate patient-reported outcomes–such as emotional well-being, acute and chronic pain, and quality of life–into routine practice to ensure a truly patient-centered approach.	5 (17)	17 (59)	3 (10)	4 (14)	0	29
34. Psychological counselling is available at hemophilia centers in Italy across all stages of the patient's life, with particular attention to adolescence and the management of treatment-related anxiety.	1 (3.5)	4 (14)	11 (38)	12 (41)	1 (3.5)	29
35. Genetic and reproductive counselling is offered at every center to women carrying a hemophilia-causing mutation and to the families of pediatric patients, from the time of diagnosis.	4 (14)	12 (41.5)	7 (24)	5 (17)	1 (3.5)	29
36. In women with abnormal uterine bleeding without documented gynecologic causes, screening is performed for inherited coagulation defects and for hemophilia carrier status, even in the absence of a family history.	7 (24)	11 (38)	5 (17)	6 (21)	0	29
37. Measurement of FVIII and FIX levels should be performed in women who are asymptomatic carriers or suspected carriers.	17 (59)	12 (41)	0	0	0	29
38. In Italian hemophilia centers, educational counselling programs for patients, caregivers, and parents of pediatric patients are an integral part of care to enhance disease understanding, promote self-management, support safe home treatment, and improve family quality of life.	7 (24)	13 (45)	7 (24)	1 (3.5)	1 (3.5)	29
